# Thiazide Diuretics and Fracture Risk: A Systematic Review and Meta‐Analysis of Randomized Clinical Trials

**DOI:** 10.1002/jbm4.10683

**Published:** 2022-10-07

**Authors:** Louis‐Charles Desbiens, Nada Khelifi, Yue‐Pei Wang, Felix Lavigne, Véronique Beaulieu, Aboubacar Sidibé, Fabrice Mac‐Way

**Affiliations:** ^1^ CHU de Québec Research Center L'Hôtel‐Dieu de Québec Hospital Quebec Canada; ^2^ Department of Medicine, Faculty of Medicine Laval University Quebec Canada

**Keywords:** BONE MINERAL DENSITY, DIURETICS, FRACTURE, SYSTEMATIC REVIEW, THIAZIDE

## Abstract

Thiazide diuretics are commonly used antihypertensive agents. Until today, whether their use reduces fracture risk remains unclear. Our objective was to conduct a systematic review of thiazide diuretics’ effects on fractures and bone mineral density (BMD) in randomized clinical trials (RCT) of adults. MEDLINE, EMBASE, CENTRAL, and the WHO's ICTRP registry were searched from inception to July 31, 2019. Two reviewers assessed studies for eligibility criteria: (i) RCTs; (ii) including adults; (iii) comparing thiazides, alone or in combination; (iv) to placebo or another medication; and (v) reporting fractures or BMD. Conference abstracts and studies comparing thiazides to antiresorptive or anabolic bone therapy were excluded. Bias was assessed using Cochrane Collaboration's Risk of Bias Tool‐2. The primary outcome was fracture at any anatomical site. Secondary outcomes were osteoporotic fractures, hip fractures, and BMD at femoral neck, lumbar spine, and/or total hip. Fractures were pooled as risk ratios (RRs) using random‐effect models. Prespecified subgroup analyses and post hoc sensitivity analyses were conducted. From 15,712 unique records screened, 32 trials (68,273 patients) met eligibility criteria. Thiazides were associated with decreased fractures at any site (RR = 0.87, 95% confidence interval [CI] 0.77–0.98; *I* ^*2*^ = 0%) and osteoporotic fractures (RR = 0.80; 95% CI 0.69–0.94; *I* ^*2*^ = 0%). Results were consistent in most subgroups and sensitivity analyses. Few studies reported hip fractures, and no association was found between thiazides and this outcome (RR = 0.84; 95% CI 0.67–1.04; *I* ^*2*^ = 0%). Only four studies reported BMD; a meta‐analysis was not conducted because BMD reporting was inconsistent. Trials were deemed at low (3 studies, weight = 3%), some concerns (16 studies; 71%), or high (11 studies; 26%) risk of bias for the primary outcome. In conclusion, thiazide diuretics decreases the risk of fractures at any and at osteoporotic sites in a meta‐analysis of RCTs. Additional studies are warranted in patients with high fracture risk. © 2022 The Authors. *JBMR Plus* published by Wiley Periodicals LLC on behalf of American Society for Bone and Mineral Research.

## Introduction

Hypertension affects 28% to 44% of individuals in occidental countries^(^ ^1^, ^2^ ^)^ and is the most important risk factor for global disease burden.^(^ ^3^ ^)^ Several pharmacological classes are recommended as first‐intention treatments for hypertension.^(^ ^4^, ^5^, ^6^ ^)^ Among these, thiazide diuretics represent 24% to 30% of antihypertensives drugs prescribed in the United States.^(^ ^7^, ^8^ ^)^ Their role in cardiovascular protection has been demonstrated in large clinical trials and meta‐analyses.^(^ ^9^, ^10^, ^11^ ^)^

Since thiazide diuretics have been associated with an enhancement of osteoblast activity and decreased calciuria in animal models and humans,^(^ ^12^, ^13^, ^14^ ^)^ it has been suggested that thiazide use could decrease fracture incidence, a major source of increased mortality, institutionalization, health costs, and decreased quality of life in aging populations.^(^ ^15^, ^16^, ^17^, ^18^ ^)^ Several epidemiological studies have indeed reported an association between thiazide use and decreased fracture risk, but these studies were limited by potential indication biases.^(^ ^19^, ^20^, ^21^, ^22^, ^23^, ^24^, ^25^ ^)^ In contrast, although no randomized controlled trial (RCT) was specifically designed to evaluate the effects of thiazides on fractures, several trials have reported fractures as adverse events.^(^ ^26^, ^27^, ^28^ ^)^ Previous systematic reviews conducted on this matter mostly focused on observational studies and did not include these RCTs.^(^ ^29^, ^30^, ^31^ ^)^ Consequently, the clinical impact of thiazide use on bone outcomes remains unclear. Because the selection of an antihypertensive agent is based on its risk–benefit profile, an enhanced appreciation of thiazides' effects on fractures would help clinicians better individualize treatment to patients' own condition.

Therefore, our primary objective was to conduct a systematic review and meta‐analysis to evaluate the effect of thiazide diuretics on fracture risk from RCTs of adult patients. Our secondary objective was to evaluate the effect of thiazide diuretics on bone mineral density (BMD).

## Methods

### Design

This study was conducted according to Cochrane and Preferred Reporting Items for Systematic Reviews and Meta‐Analysis (PRISMA) recommendations.^(^ ^32^, ^33^ ^)^ The study protocol was prospectively registered in PROSPERO (CRD42018078083).

### Search strategy

We searched MEDLINE, EMBASE, Cochrane Central Register of Randomized Clinical Trial (CENTRAL), and the World Health Organization's International Clinical Trials Registry Platform (ICTRP) databases from inception to July 31, 2019. A two‐prong strategy has been developed to identify all RCTs investigating thiazides with maximal sensibility (Supplemental Table S1). The first prong (thiazides) was composed of free text, Medical Subject Heading, and Emtree key words based on a previous Cochrane systematic review.^(^ ^29^ ^)^ The second prong (RCTs) employed the sensitivity‐ and precision‐maximizing version of the Cochrane Strategy for MEDLINE^(^ ^32^ ^)^ and a sensitivity maximizing filter with increased specificity developed for EMBASE.^(^ ^34^ ^)^ References of included studies were reviewed to identify eligible studies.

### Eligibility criteria

Included studies met the following criteria: (i) randomized clinical trials; (ii) with at least one arm including thiazide or thiazide‐like diuretics, alone or in combination; (iii) compared with placebo or non‐thiazide medications; (iv) including adults (>80% of patients); and (v) reporting fractures and/or BMD at femoral neck, total hip, or lumbar spine. Co‐interventions were allowed. Exclusion criteria were: (i) comparison of thiazides to antiresorptive agents or bone anabolic therapies and (ii) conference abstracts. No language restriction was applied.

### Study selection

Two independent reviewers assessed the eligibility of studies in a two‐step procedure. Citations' titles and abstracts were first screened. Potentially eligible studies without mention of fractures or BMD in the abstract were kept to increase the sensitivity. Full texts were then assessed to confirm eligibility. Discordances between the reviewers were solved by discussion. For duplicate reports of the same trial, the report with the most useful information was kept and the remaining reports were used as necessary. Review of trials in languages other than English or French was conducted after translation by collaborators or translators.

### Data collection

Data were extracted by two independent reviewers (LCD, FM) on a standardized form with discordances solved by discussion. A pilot extraction form was tested beforehand. The following data were collected: (i) methodology (design, follow‐up, sample size, randomization, prespecified outcomes, bias); (ii) population characteristics (age, sex, comorbidities, baseline blood pressure [BP]); (iii) interventions (medication, co‐interventions); (iv) outcome measurement (fracture assessment and validation, BMD units, and anatomical sites); and (v) outcomes (number of fractures, anatomical sites, BMD, obtained BP). Fracture events for the longest follow‐up period were collected. Post‐intervention BP was collected at the median follow‐up. Comparator groups were categorized as placebo (including no medication), non‐thiazide antihypertensives, or both/other (either non‐antihypertensive comparator or two comparator groups). Studies' authors were contacted for supplemental data as necessary. Risk of bias was assessed by two independent reviewers (LCD, FM) using the Cochrane Collaboration's Risk of Bias Tool (version 2).^(^ ^35^ ^)^

### Outcomes

The primary outcome was fracture at any anatomical site. Secondary outcomes were osteoporotic fractures,^(^ ^36^ ^)^ hip fractures, and BMD at the femoral neck, total hip, and/or lumbar spine. These BMD sites were chosen a priori because they are recommended for osteoporosis diagnosis.^(^ ^37^ ^)^

### Data synthesis

Descriptive characteristics of included studies are presented using proportions for nominal data, means or medians for continuous data. Meta‐analyses were conducted with random effects models. Fractures were pooled as risk ratios (RR) using the Mantel–Hantzel method and BMD as mean differences (MD) using the inverse variance method. Heterogeneity was assessed using the DerSimonian–Laird estimator. BMD reported as post‐intervention values in each treatment group were transformed as absolute change from baseline values using formulas provided in the Cochrane Handbook.^(^ ^32^ ^)^ Absolute (g/cm^2^) and relative (percentage) changes from baseline BMD values were pooled separately. BMD at different anatomical sites was also pooled separately. In studies with zero‐cell counts, a fixed value of 0.5 was added to each cell.^(^ ^32^ ^)^ Meta‐analysis was deemed appropriate a priori if three or more studies reported an outcome. The meta package was used in R 4.1.0 (R Foundation for Statistical Computing) to carry analyses.

### Heterogeneity and publication bias

Heterogeneity was measured with the *I* ^*2*^ statistic and categorized according to existing guidelines.^(^ ^38^ ^)^ Potential sources of heterogeneity were investigated in preplanned subgroup analyses. These analyses were performed for age, sex, baseline BP, follow‐up length, comparators, fracture reporting (outcome, adverse events), and overall risk of bias. Post hoc sensitivity analyses were conducted by excluding the results of trials restricted to some anatomical fracture sites or with a major weight in meta‐analyses. Post hoc sensitivity analyses using alternative methods for zero‐cell correction and heterogeneity estimation were conducted according to previous recommendations.^(^ ^32^, ^39^, ^40^, ^41^, ^42^, ^43^ ^)^ Publication bias was assessed by visual analysis of funnel plots and computation of Egger and Begg tests for outcomes with more than 10 studies.^(^ ^44^, ^45^, ^46^ ^)^

## Results

### Study selection and characteristics

**Fig. 1:**
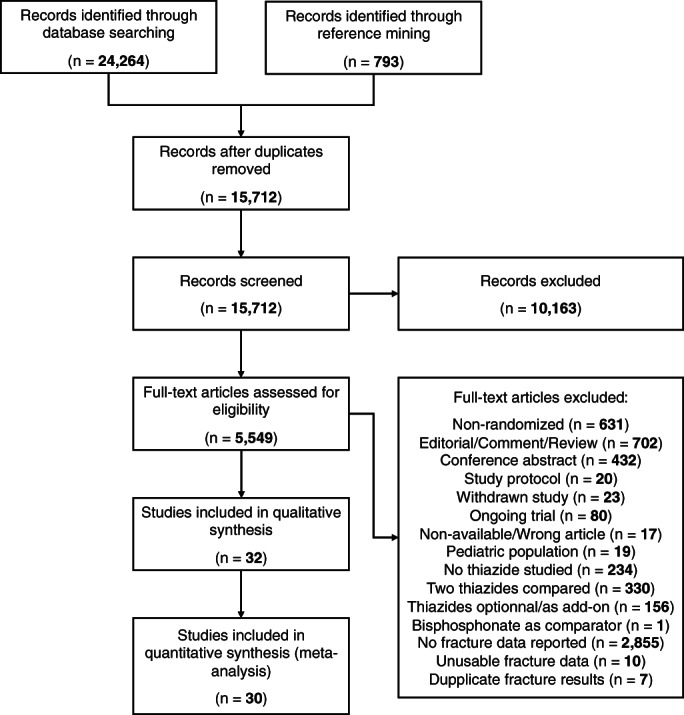
PRISMA flow diagram of study selection. PRISMA = Preferred Reporting Items for Systematic Reviews and Meta‐analyses.

From 25,057 reports retrieved, 32 studies (Table 1; 68,273 patients)^(^ ^26^, ^27^, ^28^, ^47^, ^48^, ^49^, ^50^, ^51^, ^52^, ^53^, ^54^, ^55^, ^56^, ^57^, ^58^, ^59^, ^60^, ^61^, ^62^, ^63^, ^64^, ^65^, ^66^, ^67^, ^68^, ^69^, ^70^, ^71^, ^72^, ^73^, ^74^, ^75^ ^)^ met all inclusion criteria after full‐text screening (Fig. 1). Additional data from five trials were obtained through authors' contact.^(^ ^26^, ^47^, ^52^, ^58^, ^73^ ^)^ Eight studies (10 reports)^(^ ^76^, ^77^, ^78^, ^79^, ^80^, ^81^, ^82^, ^83^ ^)^ reported fractures but were not included because it was impossible to analyze their fracture data (notably because fractures could not be attributed to a specific intervention group). Aggregated characteristics are presented in Table 2. The number of patients in studies ranged from 32 to 22,180 with a mean age of 34 to 84 years. Forty‐six percent of participants were women, with two studies conducted exclusively in women. Follow‐up duration ranged from 4 weeks to 5.6 years. As comparators, 11 trials used a placebo or medications that were also included in the thiazide arm, 12 trials used antihypertensives other than the ones given in the thiazide arm, and nine trials used both or included non‐antihypertensive medication (ertuglifozin, ibopamine, calcium, and vitamin D).

**Table 1:** Characteristics of Trials Included in the Meta‐Analysis

Study acronym/ID (country)	Population	Age (years)	Women (%)	Baseline BP (mmHg)	Duration	Thiazide arm(s)			Comparator arm(s)			Outcome(s)
						Protocol (daily doses)	*n*	Obtained BP (mmHg)	Protocol (daily doses)	*n*	Obtained BP (mmHg)	
Ando^(^ ^54^ ^)^ 2009 ONEAST (Japan)	20–80 years HTN	66	56	151/83	12 weeks	HCTZ 12.5 mg Telmisartan 40–80 mg	37	133/77	Amlodipine 5–7.5 mg	38	144/81	FX (AE)
Brown^(^ ^48^, ^92^ ^)^ 2016 *PATHWAY‐3* (UK)	18–80 years HTN 1 MetS component	62	41	155/91	24 weeks	HCTZ 25‐50 mg	146	136/NR	Amiloride 10–20 mg	145	135/NR	FX (SAE)
						HCTZ 12.5‐25 mg Amiloride 5‐10 mg	150	133/NR				
Canter^(^ ^55^ ^)^ 1994 (multinational)	>18 years HTN	53	37	162/105	8 weeks	HCTZ 6.25‐25 mg	88	‐6/−7^a^	Placebo	27	‐3/−3^a^	FX (SAE)
						HCTZ 6.25‐25 mg Quinapril 2.5–40 mg	259	−14/−11^a^	Quinapril 2.5–40 mg	86	−9/−8^a^	
Daiichi Sankyo^(^ ^73^, ^93^ ^)^ 2009 *CS8635‐A‐E303* (multinational)	>18 years HTN	56	42	148/94	8 weeks	HCTZ 12.5 mg Olmesartan 40 mg Amlodipine 10 mg	269	139/86	Olmesartan 40 mg Amlodipine 10 mg	269	140/87	FX (SAE)
						HCTZ 25 mg Olmesartan 40 mg Amlodipine 10 mg	270	136/85				
Diehm^(^ ^49^ ^)^ 2011 (multinational)	>40 years HTN PAD	66	23	148/84	24 weeks	HCTZ 25 mg	86	140/80	Nebivolol 5 mg	91	140/80	FX (SAE)
Fletcher^(^ ^50^, ^94^ ^)^ 1991 *EWPHE* (multinational)	>60 years HTN	72	70	182/101	Mean 4.6 years	HCTZ 25‐50 mg Triamterene 50−100 mg	416	150/85	Placebo	424	171/95	FX (AE)
Genthon^(^ ^56^ ^)^ 1994 (multinational)	18–75 years HTN	55	49	168/102	8 weeks	HCTZ 12.5 mg	220	149/91	Ramipril 2.5 mg	218	150/89	FX (drug‐related SAE)
						HCTZ 12.5 mg Ramipril 2.5 mg	222	148/88				
Giles^(^ ^51^, ^95^ ^)^ 1992 (USA)	HTN	63	16	135/NR	1 year	HCTZ 50 mg	15	123/NR	Nitrendipine 20‐30 mg	17	113/NR	BMD (LS)
Kario^(^ ^57^, ^96^ ^)^ 2017 *NOCTURNE* (Japan)	30–85 years HTN	63	45	146/87	8 weeks	Trichlormethiazide 1 mg Irbesartan 100 mg	208	134/82	Amlodipine 5 mg Irbesartan 100 mg	203	129/78	FX (SAE)
Kato^(^ ^58^ ^)^ 2011 *MOTHER* (Japan)	65–85 years HTN CVD risk factor	73	39	160/87	24 weeks	TZD^b^ Olmesartan	32	136/73	CCB^b^ Olmesartan	33	133/72	FX (AE)
Kleber^(^ ^59^ ^)^ 1990 (Germany)	CHF NYHA 1–2	66	66	NR	8 weeks	HCTZ 25 mg	60	NR	Placebo	63	NR	FX (AE‐withdrawal)
						HCTZ 25 mg Ibopamine 200 mg	63	NR	Ibopamine 200 mg	61	NR	
LaCroix^(^ ^28^ ^)^ 2000 (USA)	60–79 years No HTN BMD Z‐score − 2 to +2	68	63	125/NR	3 years	HCTZ 12.5 mg	108	NR	Placebo	105	NR	FX (prespecified outcome: questionnaire + radiological validation) BMD (LS, TH)
						HCTZ 25 mg	107	NR				
Lee^(^ ^60^ ^)^ 2012 (Taïwan)	20–80 years HTN Type 2 diabetes Microalbuminuria	60	41	141/87	16 weeks	HCTZ 7.25‐25 mg Valsartan 40‐160 mg	85	123/79	Amlodipine 2.5–10 mg Benazepril 5‐20 mg	84	126/78	FX (SAE)
Lonn^(^ ^61^, ^97^ ^)^ 2016 *HOPE‐3* (multinational)	M > 55 years (+ one CVD risk factor) F > 65 years (+ one CVD risk factor) F 60–65 years (+ two CVD risk factors)	66	46	138/82	Median 5.6 years	HCTZ 12.5 mg Candesartan 16 mg Rosuvastatin 10 mg randomized 1: 1	6356	129/75	Placebo Rosuvastatin 10 mg randomized 1: 1	6349	134/78	FX (hospitalization cause)
Mallion^(^ ^62^ ^)^ 2000 (France)	18–75 years HTN	56	52	163/101	12 weeks	Indapamide 0.625 mg Perindopril 2 mg	222	143/86	Atenolol 50 mg	224	143/84	FX (drug‐related SAE)
Merck^(^ ^74^, ^98^, ^99^ ^)^ 2010 *MK‐8835‐032* (multinational)	18–65 years HTN Uncontrolled type 2 diabetes	54	32	136/84	4 weeks	HCTZ 12.5 mg	39	135/83	Placebo	39	136/86	FX (SAE)
									Ertuglifozin 1‐25 mg	116	131/82	
Moser^(^ ^63^ ^)^ 1992 (USA, Bahamas)	HTN	50	34	152/102	4 weeks	HCTZ 25 mg	33	NR/94	Placebo	31	NR/97	FX (AE‐withdrawal)
									Benazepril 2.5–10 mg	142	NR/96	
Novartis^(^ ^71^, ^100^, ^101^ ^)^ 2003 *ACCOMPLISH* (multinational)	>55 years HTN CVD/target organ damage	68	40	145/80	Mean 3 years	HCTZ 12.5–25 mg Benazepril 20–40 mg	5762	131/73	Amlodipine 5–10 mg Benazepril 20‐40 mg	5744	130/71	FX (SAE)
Novartis^(^ ^72^, ^102^, ^103^ ^)^ 2008 *ACQUIRE* (multinational)	HTN >18 year	57	50	167/95	12 weeks	HCTZ 25 mg Aliskiren 300 mg	349	−30/−13^a^	Aliskiren 300 mg	339	−20/−8^a^	FX (SAE)
Novartis^(^ ^75^, ^104^, ^105^ ^)^ 2008 *ValVET* (Canada, USA)	>70 years HTN	78	56	165/85	4 weeks	HCTZ 12.5 mg	128	151/82	Valsartan 160 mg	128	158/81	FX (SAE)
						HCTZ 12.5 mg Valsartan 160 mg	128	147/78				
Perez‐Castrillon^(^ ^52^ ^)^ 2003 (Spain)	36–76 years HTN	59	60	157/93	1 year	HCTZ 12.5 mg Quinapril 40 mg	45	143/86	Quinapril 40 mg	46	138/86	BMD (LS)
									Enalapril 20 mg	43	142/88	
Peters^(^ ^27^, ^106^, ^107^, ^108^ ^)^ 2010 *HYVET* (multinational)	HTN >80 years	84	60	173/91	Mean 2.1 years	Indapamide 1.5 mg Perindopril 2–4 mg	1933	144/78	Placebo	1912	159/84	FX (prespecified outcome: SAE + systematically collected + validated with documentation)
Puttnam^(^ ^11^, ^26^, ^109^ ^)^ 2017 *ALLHAT* (multinational)	>55 years HTN 1 or more CHD risk factor	70	43	147/83	Mean 4.9 years	Chlorthalidone 12.5‐25 mg	10,174	134/75^c^	Amlodipine 2.5–10 mg	12,006	135/75^c^	Pelvic/hip FX (post‐specified outcome using administrative databases)
									Lisinopril 10–40 mg		136/75^c^	
Rakugi^(^ ^64^, ^110^ ^)^ 2015 (Japan)	20–80 years HTN	55	23	150/96	8 weeks	HCTZ 12.5 mg Losartan 50 mg Amlodipine 5 mg	164	136/86	Losartan 50 mg Amlodipine 5 mg	163	140/88	FX (SAE)
Raveau‐Landon^(^ ^65^ ^)^ 1991 (France)	>70 years HTN	83	82	181/151	24 weeks	HCTZ 25‐50 mg Amiloride 2.5–5 mg	60	146/82	Felodipine 5–20 mg	59	156/84	FX (SAE)
Reid^(^ ^47^, ^111^ ^)^ 2000 (New Zeland)	Postmenopausal F <75 years	63	100	132/82	2 years	HCTZ 50 mg	92	122/80	Placebo	93	126/82	FX (prespecified outcome: Systematically collected + radiological validation) BMD (FN, LS)
Rodgers^(^ ^66^, ^112^ ^)^ 2011 *PILL Pilot* (multinational)	>18 years FRS >7.5% FRS >5% + 2 CVD risk factors	61	20	134/80	12 weeks	HCTZ 12.5 mg Aspirin 75 mg Losartan 50 mg Amlodipine 5 mg	189	−10/−5 versus control group	Placebo	189		FX (SAE)
Saruta^(^ ^67^, ^113^, ^114^ ^)^ 2015 *COLM* (Japan)	65–85 years HTN CVD or CVD risk factor	74	48	158/87	Median 3.3 years	Low‐dose TZD^b^ Olmesartan 5–40 mg	2573	133/74	CCB^b^ Olmesartan 50–40 mg	2568	133/73	FX (SAE)
SHEP Research Group^(^ ^53^, ^115^, ^116^ ^)^ 1991 *SHEP* (multinational)	>60 years HTN	72	57	170/77	Mean 4.5 years	Chlortalidone 12.5‐25 mg ± Atenolol 25 mg, ± Reserpine 0.05 mg	2365	143/67	Placebo	2371	155/71	FX (AE)
Weidler^(^ ^68^ ^)^ 1995 (USA)	>50 years HTN	61	50	152/99	8 weeks	Indapamide 1.25 mg	111	140/91	Placebo	111	150/95	FX (withdrawal)
Weissel^(^ ^69^ ^)^ 1990 (Switzerland, Austria)	60–75 years HTN	66	55	170/101	8 weeks	HCTZ 12.5–25 mg	68	+5/+4 versus control group	Felodipine 5–10 mg	66		FX (AE‐withdrawal)
Yamada^(^ ^70^ ^)^ 1989 (Japan)	Premenopausal F Connective tissue disease Chronic prednisolone use (5 mg ID x 6 months)	34	100	NR	2 years	Trichlormethiazide 4 mg 1‐alpha‐hydroxy‐vitamin D3 0.75 μg Calcium lactate 3 g	11	NR	No medication	13	NR	Vertebral FX (prespecified outcome: systematic radiological assessment)
									1‐alpha‐hydroxy‐vitaminD3 0.75 μg Calcium lactate 3 g	14	NR	

^a^ Versus baseline.

^b^ At the investigator choice.

^c^ This data was gathered from the ALLHAT original report,^(^ ^11^ ^)^ which included more patients than the report collecting fractures.

AE = adverse event; BMD = bone mineral density; BP = blood pressure; CHD = coronary heart disease; CHF = chronic heart failure; CVD = cardiovascular disease; DBP = diastolic blood pressure; F = female; FN = femoral neck; FRS = Framingham risk score; FX = fracture; HCTZ = hydrochlorothiazide; HTN = hypertension; ID = daily; LS = lumbar spine; MetS = metabolic syndrome; Mo = months; NR = non reported; NYHA = New York Heart Association classification; PAD = peripheral artery disease; SAE = serious adverse event; SBP = systolic blood pressure; TH = total hip.

**Table 2:** Aggregated Characteristics of Included Trials

Characteristics	No. of trials	No. of patients
Patient number		
>1000 patients	6	54,927
<1000 patients	26	13,346
Age		
<50 years	1	38
50–65 years	16	6172
>65 years	15	62,063
Sex		
>75% female	3	342
25–75% female	26	67,049
<25% female	3	882
Follow‐up		
>1 year	12	61,662
<1 year	20	6611
Baseline systolic BP		
<140 mmgHg	6	13,814
140–160 mmHg	13	41,797
160–180 mmHg	9	11,418
>180 mmHg	2	959
Thiazide		
Hydrochlorothiazide	23	31,189
Chlorthalidone	2	26,916
Indapamide	3	4513
Trichlormethiazide	2	449
Investigator‐chosen	2	5206
Comparator		
Placebo	11	25,054
No medication	8	23,231
Medication used in the thiazide group	3	1823
Other antihypertensive (not used in the thiazide group)	12	40,455
Both/Other	9	2764
Fracture reporting		
Outcome	5	26,568
Prespecified	4	4388
Post‐specified	1	22,180
Adverse event	25	41,539
BMD reporting		
Lumbar spine	4	671
Femoral neck	1	185
Total hip	1	320

BP = blood pressure; BMD = bone mineral density.

### Thiazide diuretics and fractures

**Fig. 2:**
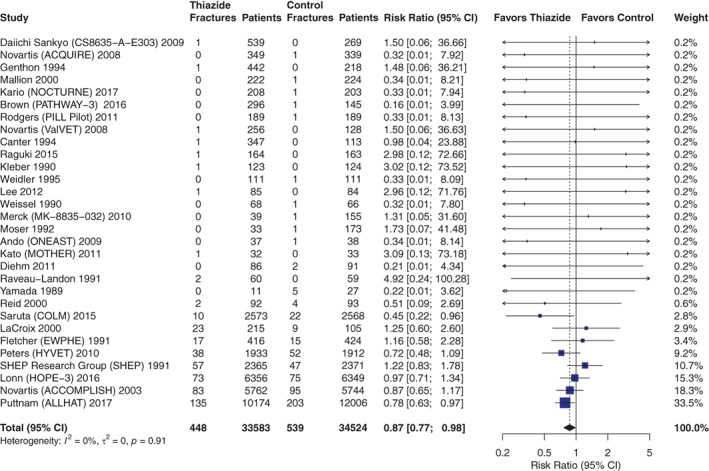
Thiazide diuretics and fractures at any anatomical site. Fractures at any anatomical sites were pooled as risk ratios (indicated as blue boxes) with 95% confidence intervals (indicated as black lines) from a random effect model. The size of the box represents the weight attributed to each study in the meta‐analysis. CI = confidence interval.

Thirty trials reporting fractures (68,107 participants; 987 fractures) were included in the meta‐analysis. Four trials reported fractures as a prespecified outcome with clinical or radiological validation and one trial reported fractures as a post‐specified outcome using medico‐administrative data. The remaining 25 trials reported fractures as adverse events. In the meta‐analysis, we observed a statistically significant decrease in fracture incidence with thiazide diuretics use (RR = 0.87 [0.77, 0.98]; *p* = 0.026; *I* ^*2*^ = 0%; Fig. 2). Eight trials had weights above 1% and totalized 96.1% of the cumulative effect.

**Fig. 3:**
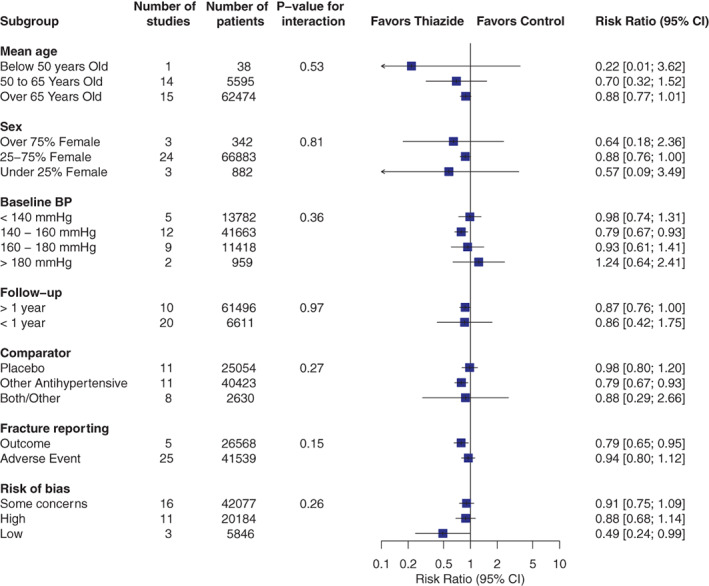
Subgroup analyses for the primary outcome. Fractures at any anatomical site were pooled as risk ratios (indicated as blue boxes) with 95% confidence intervals (indicated as black lines) from random effect models. CI = confidence interval.

In subgroup analyses (Fig. 3), we did not observe significant interactions in any prespecified subgroups. Effect estimates in subgroups were mostly similar to the overall one, except for two subgroups (age <50 years and baseline BP >180 mmHg) that included very few studies with broad confidence intervals. As a post hoc sensitivity analysis, we assessed the influence of the ALLHAT trial by removing it from the meta‐analysis and observed similar effect estimate (RR = 0.91 [0.78, 1.06]), but with an expected higher uncertainty. Removing the two trials that included anatomical fracture site restrictions (ALLHAT, hip and pelvis fractures only; Yamada and colleagues, vertebral fractures only) led to similar results (RR = 0.92 [0.79, 1.07]). Sensitivity analyses for methodological considerations (zero‐cell correction, heterogeneity estimation) also led to results similar to the principal analysis (Supplemental Table S2).

Twenty‐three trials specified anatomical sites for fractures and 22 trials (43,874 participants; 627 fractures) reported at least one osteoporotic fracture. In the meta‐analysis, thiazide diuretics significantly decreased the incidence of osteoporotic fractures (RR = 0.80 [0.69, 0.94]; *p* = 0.006; *I* ^*2*^ = 0%; Supplemental Fig. S1). Three studies had weights above 1% and totalized 94.7% of the weight. In subgroup analyses (Supplemental Fig. S2), no significant interaction was observed, whereas results were similar to the main analysis, except for two subgroups that included only one trial. Similar results were also obtained after removal of ALLHAT study (RR = 0.82 [0.66, 1.03]), removal of two studies with fracture sites restrictions (RR = 0.83 [0.66, 1.04]), and when using alternative zero‐cell correction and heterogeneity estimation methods (Supplemental Table S2).

Finally, six trials (34,814 participants; 318 fractures) reported hip fractures. In the meta‐analysis, thiazide diuretics did not significantly reduce hip fracture risk (RR = 0.84 [0.67, 1.04]; *p* = 0.116; *I* ^*2*^ = 0%; Supplemental Fig. S3). Two studies accounted for more than 98% of the weight in this meta‐analysis. Thus, subgroup and sensitivity analyses were not performed.

### Thiazide diuretics and bone mineral density

Four studies (671 participants) reported BMD: one at the femoral neck, four at the lumbar spine, and one at the total hip. Among the four studies reporting lumbar spine BMD, two used post‐intervention absolute values (g/cm^2^), while two used percent change from baseline. Therefore, no meta‐analysis was conducted (Supplemental Fig. S4). Two trials reported a significant increase in BMD for at least one anatomical site (LaCroix, total hip^(^ ^28^ ^)^; Giles, lumbar spine^(^ ^51^ ^)^), while the two other trials^(^ ^47^, ^52^ ^)^ reported a nonsignificant trend toward increased BMD with thiazide use.

### Bias assessment

**Fig. 4:**
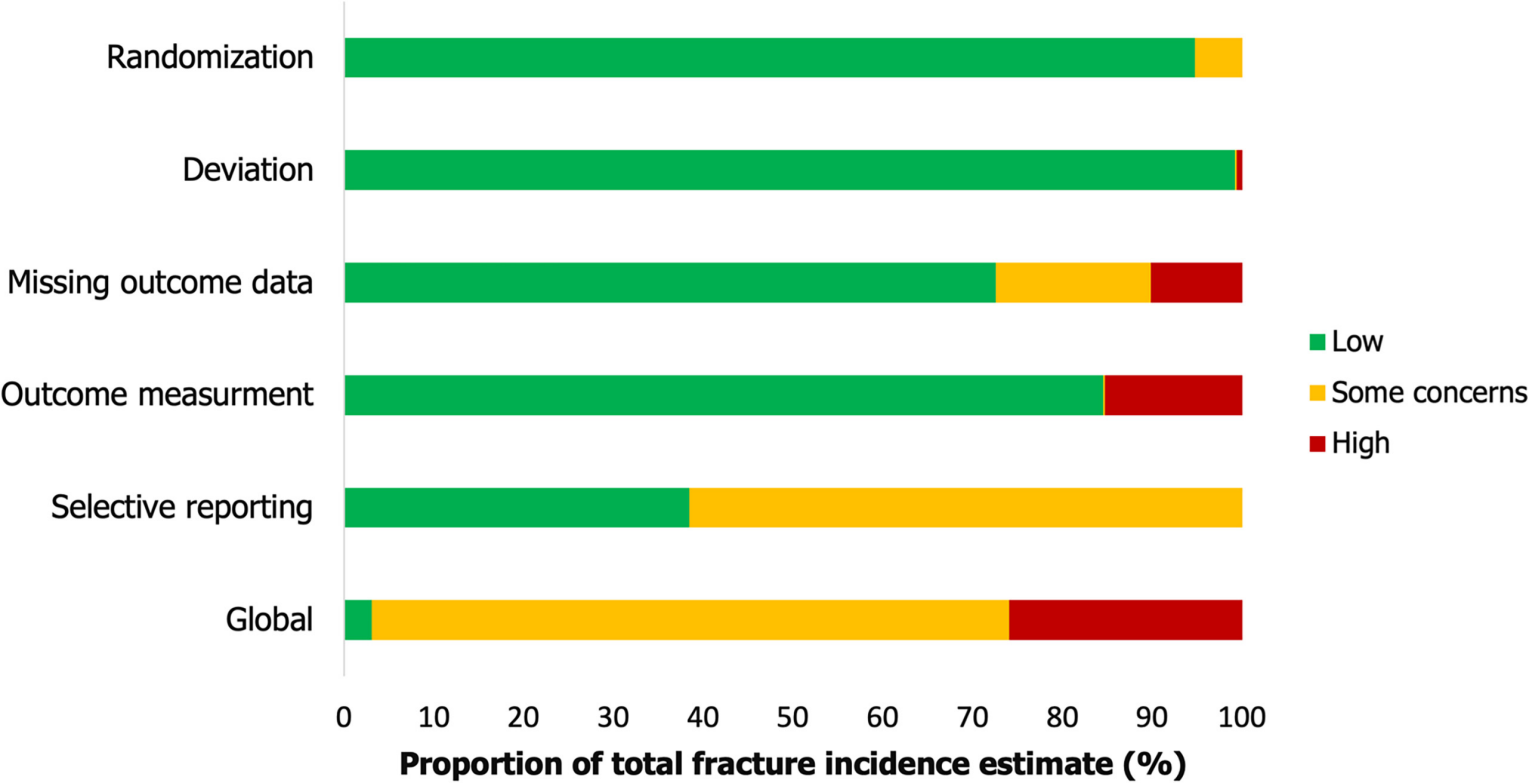
Bias assessment for the primary outcome. Risk of bias for each study was assessed using the Cochrane Collaboration's Risk of Bias tool (version 2). Bias is represented as a proportion of the weight in the primary outcome meta‐analysis.

For the primary outcome, three studies (3% of the total weight) had low risk of bias, 16 studies (71%) had some concerns, and 11 studies (26%) had high risk of bias (Fig. 4; Supplemental Table S3). Most studies were deemed at low risk in all bias domains except for selective reporting, in which 23 studies (62% of the weight) had at least some concerns for bias. For BMD, one study was judged at low risk of bias, while three studies had some concerns for bias (Table S4).

Visual analysis of funnel plots (Supplemental Fig. S5) for both any and osteoporotic fractures revealed no asymmetry suggestive of publication bias. Egger and Begg tests for funnel plot asymmetry were nonsignificant for both any (Egger *p* = 0.789; Begg *p* = 0.735) and osteoporotic fractures (Egger *p* = 0.869; Begg *p* = 0.324). Publication bias was not assessed for hip fractures and BMD considering the very small number of studies.

## Discussion

In this systematic review and meta‐analysis of RCTs, thiazide diuretics decreased the incidence of fractures when compared with placebo or other antihypertensives. Consistent results were obtained in prespecified subgroups and in post hoc analyses. Although thiazides did not specifically reduce hip fractures, very few trials reported this outcome. Meta‐analysis was not conducted for BMD considering the heterogeneity in the four studies retrieved.

Several meta‐analyses have previously investigated the effect of thiazides on fractures,^(^ ^29^, ^30^, ^31^, ^84^, ^85^ ^)^ but only one included RCTs.^(^ ^84^ ^)^ This meta‐analysis included both observational (cohort, case–control), and randomized studies. Although the authors observed a reduction in fractures (RR = 0.87 [0.70, 0.99]) similar to ours in one of their analyses, they used “fracture” in their search strategy and hence only found two RCTs (HYVET^(^ ^27^ ^)^; ALLHAT^(^ ^26^ ^)^) that were not pooled together. In contrast, the other trials found in our review did not include “fracture” in their abstract and were therefore not retrieved in that previous systematic review. Similarly, although two previous meta‐analyses were restricted to observational studies,^(^ ^31^, ^85^ ^)^ two others aimed at including RCTs but did not retrieve any.^(^ ^29^, ^30^ ^)^ This might be explained by (i) a research strategy that used “fracture”; (ii) the publication of eligible trials after their completion; and (iii) a lack of research in trials registries. Despite these discrepancies, these meta‐analyses reported results close to ours. Indeed, Xiao and colleagues observed that thiazides reduced any and hip fracture risk similar to ours (RR = 0.86 and RR = 0.82).^(^ ^31^ ^)^ In a Cochrane review, Aung and colleagues also observed a similar reduction in hip fracture risk with thiazides (RR = 0.76 [0.64, 0.89]).^(^ ^29^ ^)^ Although these results from meta‐analyses of observational studies are expected as they retrieved similar studies, they reinforce our findings of reduced fractures with thiazides use in RCTs.

Two mechanisms are hypothesized to explain the bone‐protective effects of thiazides. First, thiazides decrease urinary calcium excretion and therefore increase calcium available for bone mineralization.^(^ ^12^, ^14^ ^)^ In addition, thiazides can directly stimulate bone formation by increasing osteoblast differentiation markers and decreasing osteocalcin.^(^ ^13^, ^86^, ^87^ ^)^ Although these mechanisms have been previously supported by observational studies associating thiazides to increased BMD,^(^ ^19^, ^24^, ^25^, ^88^ ^)^ meta‐analysis for BMD was not possible in this study. Indeed, we only found four studies reporting variable and inconsistent BMD results that prevented us from conducting a meta‐analysis. Nevertheless, two trials reported significantly increased BMD with thiazides at a single bone site.

Clinical heterogeneity was observed in our meta‐analysis. For example, follow‐up duration varied from 4 weeks to several years. Likewise, various comparators were used: placebo, other antihypertensives, or non‐antihypertensive medications. This variation in comparators may have influenced our results in several ways. For example, studies comparing thiazides to a placebo may have led to differences in blood pressure that could have an impact on the risk of falls. Unfortunately, data on falls are rarely reported even in studies focusing on fracture. Similarly, the potential direct impact of non‐thiazide comparators (notably beta‐blockers and SGLT2 inhibitors) on bone may also have influenced our findings. Furthermore, two types of fracture reporting were identified: (i) as an outcome through radiological validation or large medico‐administrative databases identification or (ii) as an adverse event (AE) with much fewer details (categorized as serious AEs or treatment‐related AEs). We decided a priori to avoid using arbitrary eligibility criteria or cut‐offs and preferred using prespecified subgroup analyses to explore the influence of these characteristics. Here, we did not observe any significant interaction with study characteristics in our subgroup analyses. Moreover, no statistical heterogeneity was observed in global analyses, nor in sensitivity analyses using other methods for heterogeneity estimation. Taken together, these observations reinforce our principal findings by mitigating the potential impact of clinical heterogeneity in our results.

We used the recently updated Cochrane Risk of Bias tool (RoB2) to assess potential biases in included studies. Although some trials were deemed at high risk, these only accounted for less than a third of the weight in the principal analysis. From these, only one large study (HOPE‐3) was deemed at high risk for the outcome measurement domain because it used hospitalizations for fractures as an outcome. Similarly, only one large study (HYVET) was deemed at high risk for missing outcome data due to many deaths for which fracture could not be excluded with certainty. Nevertheless, no interaction with bias was observed in subgroup analyses.

An unexpected finding of our study was the large number of potentially eligible trials that were excluded based on non‐reported fractures. Indeed, several excluded trials at the full‐text stage only reported adverse events deemed as serious or treatment‐related. Because these definitions are highly variable, several fractures that may have occurred in these studies could have been omitted. This non‐reporting bias, which is not accounted in the RoB2 tool, may have decreased the power of our review to detect the effects on rare outcomes, such as hip fractures. Hence, our results highlight the need for full AE disclosure in RCTs as their relevance may be discovered years later.

Our study has several strengths. We conducted a comprehensive literature search in several databases including a trial registry and screened a very large number of citations. By avoiding fracture‐related terms in our search strategy, including trials reporting fractures as AEs and avoiding arbitrary selection criteria, we retrieved several trials omitted by previous reviews. Similarly, we solely included RCTs to avoid biases associated with observational studies in contrast to previous reviews.

Our study also has limitations. First, some trials did not disclose fracture sites, which increased the uncertainty for secondary fracture outcomes. Second, a single trial had a major weight in our meta‐analysis, but its removal in sensitivity analyses did not influence the magnitude of the association. Third, most trials included low‐fracture‐risk patients, which may have led to underestimation of thiazides' effect magnitude, since osteoporosis treatments are known to have larger effects in higher‐risk patients.^(^ ^89^, ^90^ ^)^ Further studies should therefore be conducted in higher‐risk populations before prompt generalization could be made to these patients. Fourth, several trials included a small number of patients with short follow‐up for which the expected effect of thiazides on bone outcome is minimal. Nevertheless, there was no interaction for follow‐up duration and our results were robust after post hoc analyses exploring other corrections for zero‐cell studies. Fifth, our meta‐analysis included heterogenous trials conducted in various populations and settings. However, statistical heterogeneity was negligible (even in analyses using other heterogeneity estimators) and subgroup analyses revealed no significant interactions. Sixth, all fracture sites were considered, including fractures less related to skeletal fragility (such as skull, hand, and feet). Similarly, fracture mechanism was not provided by most studies, which prevented us from distinguishing between low‐ and high‐trauma fractures. Nevertheless, it was previously shown that the association of low BMD with low‐ and high‐trauma fractures is similar.^(^ ^91^ ^)^

In this meta‐analysis of RCTs, thiazide diuretics were associated with decreased fractures at any and osteoporotic sites when compared with placebo or other antihypertensive therapy. Thiazide diuretics were not associated with decreased hip fractures, but uncertainty was higher for this outcome. Meta‐analysis could not be conducted for BMD because of increased heterogeneity. Our review thus strengthens past meta‐analyses of observational studies while being less prone to the indication biases of these past studies. Taken together, our results thus suggest that thiazide diuretics may play a role in fracture reduction in low‐risk patients. Further studies are warranted to assess their impact in higher‐fracture risk patients.

## Disclosures

All authors state that they have no conflicts of interest.

## Author Contributions

**Louis‐Charles Desbiens:** Conceptualization; data curation; formal analysis; methodology; writing – original draft. **Nada Khelifi:** Data curation; formal analysis; methodology. **Yue Pei Wang:** Formal analysis; methodology; writing – review and editing. **Felix Lavigne:** Formal analysis; methodology; writing – review and editing. **Véronique Beaulieu:** Formal analysis; methodology; writing – review and editing. **Aboubacar Sidibe:** Methodology; writing – review and editing. **Fabrice Mac‐Way:** Conceptualization; data curation; formal analysis; methodology; writing – review and editing.

### Peer Review

The peer review history for this article is available at https://publons.com/publon/10.1002/jbm4.10683.

## Supporting information


**Appendix S1.** Supporting information

## Data Availability

The data that support the findings of this study are available from the corresponding author upon reasonable request.
