# Supplementary material for: Thiazide Diuretics and Fracture Risk: A Systematic Review and Meta‐Analysis of Randomized Clinical Trials
Source: JBMR Plus. 2022 Oct 7;6(11):e10683. doi: 10.1002/jbm4.10683 (PMC9664541; doi:10.1002/jbm4.10683)
Supplement: Supplementary file 1 — Appendix S1. Supporting information [file JBM4-6-e10683-s001.docx]

**Title: Thiazide Diuretics and Fracture Risk: A Systematic Review and Meta-Analysis of Randomized Clinical Trials**

Louis-Charles Desbiens MD MSc^1,2^, Nada Khelifi^1,2^, Yue-Pei Wang MD MSc^1,2^,
Felix Lavigne MD^1,2^, Véronique Beaulieu MD^1,2^, Aboubacar Sidibé MSc^1,2^,
Fabrice Mac-Way MD^1,2^

^1^ CHU de Québec Research Center, Quebec City, QC, Canada

^2^ Department of Medicine, Faculty of Medicine, Laval University, Quebec City, QC, Canada

Contents

[eTable 1. Search strategy 2](#_Toc105617547)

[eTable 2. Sensitivity analyses for zero-cell correction and heterogeneity estimation 6](#_Toc105617548)

[eTable 3. Detailed risk of bias assessment for the primary outcome 7](#_Toc105617549)

[eTable 4. Detailed risk of bias assessment of each study for bone mineral density 8](#_Toc105617550)

[eFigure 1. Thiazide diuretics and osteoporotic fractures 9](#_Toc105617551)

[eFigure 2. Subgroup analyses for osteoporotic fractures 10](#_Toc105617552)

[eFigure 3. Thiazide diuretics and hip fractures 11](#_Toc105617553)

[eFigure 4. Thiazide diuretics and bone mineral density 12](#_Toc105617554)

[eFigure 5. Publication bias assessment 13](#_Toc105617555)

# eTable 1. Search strategy

| **MEDLINE PubMed** | |
| --- | --- |
| 1 | bendrofluazid [tiab] OR bendrofluazide [tiab] OR bendrofluazides [tiab] OR  bendroflumethiazid [tiab] OR bendroflumethiazide [tiab] OR bendroflumethiazides [tiab] OR  benzhydroflumethiazid [tiab] OR benzhydroflumethiazide [tiab] OR benzhydroflumethiazides [tiab] OR  benzthiazid [tiab] OR benzthiazide [tiab] OR benzthiazides [tiab] OR  benzydroflumethiazid [tiab] OR benzydroflumethiazide [tiab] OR benzydroflumethiazides [tiab] OR  benzothiadiazin [tiab] OR benzothiadiazine [tiab] OR benzothiadiazines [tiab] OR  buthiazid [tiab] OR buthiazide [tiab] OR buthiazides [tiab] OR  chlortalidon [tiab] OR chlortalidone [tiab] OR chlortalidones [tiab] OR  chlorthalidon [tiab] OR chlorthalidone [tiab] OR chlorthalidones [tiab] OR  chlorothiazid [tiab] OR chlorothiazide [tiab] OR chlorothiazides [tiab] OR  chlorphthalidolon [tiab] OR chlorphthalidolone [tiab] OR chlorphthalidolones [tiab] OR  clopamid [tiab] OR clopamide [tiab] OR clopamides [tiab] OR  clopamin [tiab] OR clopamine [tiab] OR clopamines [tiab] OR  clorexolon [tiab] OR clorexolone [tiab] OR clorexolones [tiab] OR  cyclopenthiazid [tiab] OR cyclopenthiazide [tiab] OR cyclopenthiazides [tiab] OR  cyclothiazid [tiab] OR cyclothiazide [tiab] OR cyclothiazides [tiab] OR  diapamid [tiab] OR diapamide [tiab] OR diapamides [tiab] OR  diucardin [tiab] OR  diuril [tiab] OR  enduron [tiab] OR  esidrix [tiab] OR  ezna [tiab] OR  fenquizon [tiab] OR fenquizone [tiab] OR fenquizones [tiab] OR  hctz [tiab] OR  hydrochlorothiazid [tiab] OR hydrochlorothiazide [tiab] OR hydrochlorothiazides [tiab] OR  hydroflumethiazid [tiab] OR hydroflumethiazide [tiab] OR hydroflumethiazides [tiab] OR  hydromox [tiab] OR  hygroton [tiab] OR  indapamid [tiab] OR indapamide [tiab] OR indapamides [tiab] OR  isodapamid [tiab] OR isodapamide [tiab] OR isodapamides [tiab] OR  lozol [tiab] OR  mefrusid [tiab] OR mefruside [tiab] OR mefrusides [tiab] OR  methyclothiaizid [tiab] OR methyclothiaizide [tiab] OR methyclothiaizides [tiab] OR  metindamin [tiab] OR metindamine [tiab] OR metindamines [tiab] OR  metholazon [tiab] OR metholazone [tiab] OR metholazones [tiab] OR  metolazon [tiab] OR metolazone [tiab] OR metolazones [tiab] OR  microzid [tiab] OR microzide [tiab] OR microzides [tiab] OR  mykrox [tiab] OR  naqua [tiab] OR  naturetin [tiab] OR  oxodolin [tiab] OR oxodoline [tiab] OR oxodolines [tiab] OR  phthalamudin [tiab] OR phthalamudine [tiab] OR phthalamudines [tiab] OR  polythiazid [tiab] OR polythiazide [tiab] OR polythiazides [tiab] OR  quinethazon [tiab] OR quinethazone [tiab] OR quinethazones [tiab] OR  renese [tiab] OR  s-1520 [tiab] OR s1520 [tiab] OR se-1520 [tiab] OR se1520 [tiab] OR  thaliton [tiab] OR thalitone [tiab] OR thalitones [tiab] OR  thiazid [tiab] OR thiazide [tiab] OR thiazides [tiab] OR  "thiazide-like" [tiab] OR  trichlormethiazid [tiab] OR trichlormethiazide [tiab] OR trichlormethiazides [tiab] OR  xipamid [tiab] OR xipamide [tiab] OR xipamides [tiab] OR  zaroxolyn [tiab] |
|  |  |
| 2 | "Thiazides" [mesh] OR "Sodium Chloride Symporter Inhibitors"[Mesh] OR "Sodium Chloride Symporter Inhibitors" [Pharmacological Action] |
|  |  |
| 3 | #1 OR #2 |
|  |  |
| 4 | randomized controlled trial [pt] OR controlled clinical trial [pt] OR randomized [tiab] OR placebo [tiab] OR clinical trials as topic [mesh: noexp] OR randomly [tiab] OR trial [ti] |
|  |  |
| 5 | animals [mh] NOT humans [mh] |
|  |  |
| 6 | #4 NOT #5 |
|  |  |
| 7 | #3 AND #6 |

| **EMBASE** | |
| --- | --- |
| 1 | (bendrofluazid OR bendrofluazide OR bendrofluazides OR  bendroflumethiazid OR bendroflumethiazide OR bendroflumethiazides OR  benzhydroflumethiazid OR benzhydroflumethiazide OR benzhydroflumethiazides OR  benzthiazid OR benzthiazide OR benzthiazides OR  benzydroflumethiazid OR benzydroflumethiazide OR benzydroflumethiazides OR  benzothiadiazin OR benzothiadiazine OR benzothiadiazines OR  buthiazid OR buthiazide OR buthiazides OR  chlortalidon OR chlortalidone OR chlortalidones OR  chlorthalidon OR chlorthalidone OR chlorthalidones OR  chlorothiazid OR chlorothiazide OR chlorothiazides OR  chlorphthalidolon OR chlorphthalidolone OR chlorphthalidolones OR  clopamid OR clopamide OR clopamides OR  clopamin OR clopamine OR clopamines OR  clorexolon OR clorexolone OR clorexolones OR  cyclopenthiazid OR cyclopenthiazide OR cyclopenthiazides OR  cyclothiazid OR cyclothiazide OR cyclothiazides OR  diapamid OR diapamide OR diapamides OR  diucardin OR  diuril OR  enduron OR  esidrix OR  ezna OR  fenquizon OR fenquizone OR fenquizones OR  hctz OR  hydrochlorothiazid OR hydrochlorothiazide OR hydrochlorothiazides OR  hydroflumethiazid OR hydroflumethiazide OR hydroflumethiazides OR  hydromox OR  hygroton OR  indapamid OR indapamide OR indapamides OR  isodapamid OR isodapamide OR isodapamides OR  lozol OR  mefrusid OR mefruside OR mefrusides OR  methyclothiaizid OR methyclothiaizide OR methyclothiaizides OR  metindamin OR metindamine OR metindamines OR  metholazon OR metholazone OR metholazones OR  metolazon OR metolazone OR metolazones OR  microzid OR microzide OR microzides OR  mykrox OR  naqua OR  naturetin OR  oxodolin OR oxodoline OR oxodolines OR  phthalamudin OR phthalamudine OR phthalamudines OR  polythiazid OR polythiazide OR polythiazides OR  quinethazon OR quinethazone OR quinethazones OR  renese OR  s-1520 OR s1520 OR se-1520 OR se1520 OR  thaliton OR thalitone OR thalitones OR  thiazid OR thiazide OR thiazides OR  ‘thiazide-like’ OR  trichlormethiazid OR trichlormethiazide OR trichlormethiazides OR  xipamid OR xipamide OR xipamides OR  zaroxolyn):ab,ti |
|  |  |
| 2 | 'thiazide diuretic agent'/exp |
|  |  |
| 3 | #1 OR #2 |
|  |  |
| 4 | random*:ab,ti OR 'clinical trial*':ab,ti,kw OR 'treatment outcome'/exp |
|  |  |
| 5 | #3 AND #4 |

| **CENTRAL** | |
| --- | --- |
| 1 | (bendrofluazid or bendrofluazide or bendrofluazides or bendroflumethiazid or bendroflumethiazide or bendroflumethiazides or benzhydroflumethiazid or benzhydroflumethiazide or benzhydroflumethiazides or benzthiazid or benzthiazide or benzthiazides or benzydroflumethiazid or benzydroflumethiazide or benzydroflumethiazides or benzothiadiazin or benzothiadiazine or benzothiadiazines or buthiazid or buthiazide or buthiazides or chlortalidon or chlortalidone or chlortalidones or chlorthalidon or chlorthalidone or chlorthalidones or chlorothiazid or chlorothiazide or chlorothiazides or chlorphthalidolon or chlorphthalidolone or chlorphthalidolones or clopamid or clopamide or clopamides or clopamin or clopamine or clopamines or clorexolon or clorexolone or clorexolones or cyclopenthiazid or cyclopenthiazide or cyclopenthiazides or cyclothiazid or cyclothiazide or cyclothiazides or diapamid or diapamide or diapamides or diucardin or diuril or enduron or esidrix or ezna or fenquizon or fenquizone or fenquizones or hctz or hydrochlorothiazid or hydrochlorothiazide or hydrochlorothiazides or hydroflumethiazid or hydroflumethiazide or hydroflumethiazides or hydromox or hygroton or indapamid or indapamide or indapamides or isodapamid or isodapamide or isodapamides or lozol or mefrusid or mefruside or mefrusides or methyclothiaizid or methyclothiaizide or methyclothiaizides or metindamin or metindamine or metindamines or metholazon or metholazone or metholazones or metolazon or metolazone or metolazones or microzid or microzide or microzides or mykrox or naqua or naturetin or oxodolin or oxodoline or oxodolines or phthalamudin or phthalamudine or phthalamudines or polythiazid or polythiazide or polythiazides or quinethazon or quinethazone or quinethazones or renese or s-1520 or s1520 or se-1520 or se1520 or thaliton or thalitone or thalitones or thiazid or thiazide or thiazides or "thiazide-like" or trichlormethiazid or trichlormethiazide or trichlormethiazides or xipamid or xipamide or xipamides or zaroxolyn):ti,ab |
|  |  |
| 2 | MeSH descriptor: [Thiazides] explode all trees OR MeSH descriptor : [Sodium Chloride Symporter Inhibitors] explode all trees |
|  |  |
| 3 | #1 OR #2 |

| **ICTRP** | |
| --- | --- |
| 1 | (bendrofluazid or bendrofluazide or bendrofluazides or bendroflumethiazid or bendroflumethiazide or bendroflumethiazides or benzhydroflumethiazid or benzhydroflumethiazide or benzhydroflumethiazides or benzthiazid or benzthiazide or benzthiazides or benzydroflumethiazid or benzydroflumethiazide or benzydroflumethiazides or benzothiadiazin or benzothiadiazine or benzothiadiazines or buthiazid or buthiazide or buthiazides or chlortalidon or chlortalidone or chlortalidones or chlorthalidon or chlorthalidone or chlorthalidones or chlorothiazid or chlorothiazide or chlorothiazides or chlorphthalidolon or chlorphthalidolone or chlorphthalidolones or clopamid or clopamide or clopamides or clopamin or clopamine or clopamines or clorexolon or clorexolone or clorexolones or cyclopenthiazid or cyclopenthiazide or cyclopenthiazides or cyclothiazid or cyclothiazide or cyclothiazides or diapamid or diapamide or diapamides or diucardin or diuril or enduron or esidrix or ezna or fenquizon or fenquizone or fenquizones or hctz or hydrochlorothiazid or hydrochlorothiazide or hydrochlorothiazides or hydroflumethiazid or hydroflumethiazide or hydroflumethiazides or hydromox or hygroton or indapamid or indapamide or indapamides or isodapamid or isodapamide or isodapamides or lozol or mefrusid or mefruside or mefrusides or methyclothiaizid or methyclothiaizide or methyclothiaizides or metindamin or metindamine or metindamines or metholazon or metholazone or metholazones or metolazon or metolazone or metolazones or microzid or microzide or microzides or mykrox or naqua or naturetin or oxodolin or oxodoline or oxodolines or phthalamudin or phthalamudine or phthalamudines or polythiazid or polythiazide or polythiazides or quinethazon or quinethazone or quinethazones or renese or s-1520 or s1520 or se-1520 or se1520 or thaliton or thalitone or thalitones or thiazid or thiazide or thiazides or "thiazide-like" or trichlormethiazid or trichlormethiazide or trichlormethiazides or xipamid or xipamide or xipamides or zaroxolyn) |
|  |  |

# eTable 2. Sensitivity analyses for zero-cell correction and heterogeneity estimation

| **Analysis** | **Fractures at any site** | **Osteoporotic fractures** |
| --- | --- | --- |
| **Zero-cell correction** |  |  |
| Main analysis (Mantel-Hantzel with 0.5 correction) | RR=0.87 (0.77, 0.98) τ^2^ = 0, I^2^ = 0% | RR=0.80 (0.69, 0.94) τ^2^ = 0, I^2^ = 0% |
| Peto odds ratio | OR=0.86 (0.74, 1.00) τ^2^ = 0.010, I^2^ = 6% | OR=0.80 (0.68, 0.94) τ^2^ = 0, I^2^ = 0% |
| Treatment arm continuity correction | RR=0.87 (0.77, 0.98) τ^2^ = 0, I^2^ = 0% | RR=0.80 (0.69, 0.94) τ^2^ = 0, I^2^ = 0% |
| Mantel-Hantzel exact odds ratio without correction | OR=0.86 (0.76, 0.98) τ^2^ = 0, I^2^ = 0% | OR=0.80 (0.68, 0.94) τ^2^ = 0, I^2^ = 0% |
|  |  |  |
| **Heterogeneity variance estimation** |  |  |
| Main analysis (DerSimonian-Laird estimator) | RR=0.87 (0.77, 0.98) τ^2^ = 0, I^2^ = 0% | RR=0.80 (0.69, 0.94) τ^2^ = 0, I^2^ = 0% |
| Paule and Mandel method | RR=0.87 (0.77, 0.98) τ^2^ = 0, I^2^ = 0% | RR=0.80 (0.69, 0.94) τ^2^ = 0, I^2^ = 0% |
| Restricted maximum likelihood estimator | RR=0.87 (0.76, 0.99) τ^2^ = 0.003, I^2^ = 0% | RR=0.80 (0.69, 0.94) τ^2^ = 0, I^2^ = 0% |
| OR, Odds ratio; RR, Risk ratio | | |

# eTable 3. Detailed risk of bias assessment for the primary outcome

| **Study** | **Overall** | **Randomization process** | **Deviations from intended interventions** | **Missing outcome data** | **Outcome measurement** | **Selective reporting** |
| --- | --- | --- | --- | --- | --- | --- |
| **Ando 2009** | **Some concerns** | Some concerns | Low | Low | Low | Some concerns |
| **Brown 2016** | **High** | Low | Low | High | Low | Some concerns |
| **Canter 1994** | **Some concerns** | Some concerns | Low | Low | Low | Some concerns |
| **Daiichi Sankyo 2009** | **High** | Some concerns | Low | High | Low | Some concerns |
| **Diehm 2011** | **Some concerns** | Low | Low | Some concerns | Low | Some concerns |
| **Fletcher 1991** | **Some concerns** | Some concerns | Low | Some concerns | Low | Some concerns |
| **Genthon 1994** | **High** | Some concerns | Low | High | Low | Some concerns |
| **Kario 2017** | **Some concerns** | Low | Low | Some concerns | Low | Low |
| **Kato 2011** | **High** | Low | High | Low | Low | Some concerns |
| **Kleber 1990** | **High** | Some concerns | High | High | Low | Some concerns |
| **LaCroix 2000** | **Some concerns** | Low | Low | Some concerns | Low | Some concerns |
| **Lee 2012** | **Some concerns** | Low | Low | Low | Low | Some concerns |
| **Lonn 2016** | **High** | Low | Low | Low | High | Low |
| **Mallion 2000** | **Some concerns** | Some concerns | Low | Low | Low | Some concerns |
| **Merck 2010** | **Some concerns** | Some concerns | Low | Low | Low | Some concerns |
| **Moser 1992** | **Some concerns** | Some concerns | Low | Low | Low | Some concerns |
| **Novartis 2003 (ACCOMPLISH)** | **Some concerns** | Low | Low | Low | Low | Some concerns |
| **Novartis 2008  (ACQUIRE)** | **High** | Low | Low | High | Low | Some concerns |
| **Novartis 2008**  **(ValVET)** | **High** | Low | Low | High | Low | Some concerns |
| **Peters 2010** | **High** | Low | Low | High | Low | Low |
| **Puttnam 2017** | **Some concerns** | Low | Low | Low | Low | Some concerns |
| **Rakugi 2015** | **Low** | Low | Low | Low | Low | Low |
| **Raveau-Landon 1991** | **High** | Some concerns | High | Low | Low | Some concerns |
| **Reid 2000** | **Some concerns** | Low | Low | Low | Low | Some concerns |
| **Rodgers 2011** | **Low** | Low | Low | Low | Low | Low |
| **Saruta 2015** | **Low** | Low | Low | Low | Low | Low |
| **SHEP Research Group 1991** | **Some concerns** | Low | Low | Some concerns | Low | Low |
| **Weidler 1995** | **High** | Some concerns | High | Low | Low | Some concerns |
| **Weissel 1990** | **Some concerns** | Some concerns | Low | Low | Low | Some concerns |
| **Yamada 1989** | **Some concerns** | Some concerns | Some concerns | Low | Some concerns | Some concerns |

# eTable 4. Detailed risk of bias assessment of each study for bone mineral density

| **Study** | **Overall** | **Randomization process** | **Deviations from intended interventions** | **Missing outcome data** | **Outcome measurement** | **Selective reporting** |
| --- | --- | --- | --- | --- | --- | --- |
| **Giles 1992** | **Some concerns** | Some concerns | Low | Low | Low | Some concerns |
| **LaCroix 2000** | **Some concerns** | Low | Low | Low | Low | Some concerns |
| **Perez-Castrillon 2003** | **Some concerns** | Some concerns | Some concerns | Low | Low | Some concerns |
| **Reid 2000** | **Low** | Low | Low | Low | Low | Low |

# eFigure 1. Thiazide diuretics and osteoporotic fractures

Osteoporotic fractures were pooled as risk ratios (symbolized as blue boxes) with 95% confidence intervals (symbolized as black lines) from a random effect model. The size of the box represents the weight attributed to each study in the meta-analysis.

CI, Confidence interval.

# eFigure 2. Subgroup analyses for osteoporotic fractures

Osteoporotic fractures were pooled as risk ratios (symbolized as blue boxes) with 95% confidence intervals (symbolized as black lines) from random effect models.

CI, Confidence interval.

# eFigure 3. Thiazide diuretics and hip fractures

Hip fractures were pooled as risk ratios (symbolized as blue boxes) with 95% confidence intervals (symbolized as black lines) from a random effect model. The size of the box represents the weight attributed to each study in the meta-analysis.

CI, Confidence interval.

# eFigure 4. Thiazide diuretics and bone mineral density

Bone mineral density is presented as mean differences (symbolized as blue boxes) with 95% confidence intervals (symbolized as black lines). No meta-analysis was conducted. The size of the box is fixed for each study.

CI, Confidence interval.

# eFigure 5. Publication bias assessment

Publication bias assessment for the primary outcome (any fractures) and one secondary outcome (osteoporotic fractures) was conducted using standard funnel plots. Each included study is represented by a grey dot.
